# Differences in the Concentration of Vitamin D Metabolites in Plasma Due to the Low-Carbohydrate-High-Fat Diet and the Eastern European Diet—A Pilot Study

**DOI:** 10.3390/nu13082774

**Published:** 2021-08-13

**Authors:** Izabela Bolesławska, Magdalena Kowalówka, Małgorzata Dobrzyńska, Marta Karaźniewicz-Łada, Juliusz Przysławski

**Affiliations:** 1 Department of Bromatology, Poznan University of Medical Sciences, 42 Marcelińska Street, 60-354 Poznań, Poland; ibolesla@ump.edu.pl (I.B.); mkowalowka@ump.edu.pl (M.K.); mdobrzynska@ump.edu.pl (M.D.); jprzysla@ump.edu.pl (J.P.); 2 Department of Physical Pharmacy and Pharmacokinetics, Poznan University of Medical Sciences, 6 Święcickiego Street, 60-781 Poznań, Poland

**Keywords:** vitamin D, 25(OH)D_3_ concentration, low-carbohydrate-high-fat diet, Eastern European diet, nutrition

## Abstract

Vitamin D deficiency is a global problem with many health consequences, and it is currently recommended to supplement vitamin D. Change of diet should also be considered to ensure adequate vitamin D in the human body. The aim of this study was to assess the concentration of vitamin D metabolites in two different groups: one group on the low-carbohydrate-high-fat (LCHF) diet and the other group on the Eastern European (EE) diet. In the first stage, 817 participants declaring traditional EE diet or LCHF diet were investigated. Nutrition (self-reported 3-day estimated food record) and basic anthropometric parameters were assessed. After extra screening, 67 participants on the EE diet and 41 on the LCHF diet were qualified for the second stage. Plasma 25-hydroxycholecalciferol (25(OH)D_3_) and (25(OH)D_2_) concentration was measured by the validated HPLC—MS/MS method. Plasma 25(OH)D_3_ concentration was significantly higher in the group on the LCHF diet (34.9 ± 15.9 ng/mL) than in the group on the EE diet (22.6 ± 12.1 ng/mL). No statistical differences were observed in plasma 25(OH)D_2_ concentration between the study groups (*p* > 0.05). Women had a higher plasma 25(OH)D_2_ concentration than men regardless of diet type. The LCHF diet had a positive influence on plasma vitamin D concentration. However, long-term use of the LCHF diet remains contentious due to the high risk of cardiovascular disease. This study confirmed that the type of diet influences the concentration of vitamin D metabolites in the plasma.

## 1. Introduction

There are two main forms of vitamin D: cholecalciferol (vitamin D_3_) and ergocalciferol (vitamin D_2_). Vitamin D_3_ is found in animal-sourced foods (fish rich in fats, egg yolk, milk and dairy products), whereas vitamin D_2_ comes mainly from plant sources (yeast, mushrooms and fortified foods) [1,2].

A major source of vitamin D in the human body is by endogenous synthesis from 7-dehydrocholesterol in the skin’s epidermis after exposure to UV (ultraviolet) B radiation with wavelengths between 290 and 315 nm [3].

The most active metabolite of vitamin D is 1,25-dihydroxycholecalciferol (1,25(OH)_2_D_3_). It is a product of two-stage hydroxylation of 25-hydroxycholecalciferol (25(OH)D_3_) in the liver and hydroxylation in position 1α in the kidney and other organs [4,5,6]. The active form of vitamin D has effects on many organs and tissues in the human body. The pleiotropic effect of this vitamin concerns not only the calcium-phosphorus metabolism but also the immune, cardiovascular and nervous systems [7]. An epidemiological and clinical study showed that it is essential to ensure the proper intake of this vitamin.

Unfortunately, the prevalence of vitamin D deficiency is still high, especially in industrialized countries [8]. The causes of deficiency are probably related to UV filter usage, increased atmospheric pollution, excessive skin pigmentation, and ageing of the world population [9]. Moreover, the concentration of vitamin D should be analyzed taking into account the influence of factors such as seasonality and the influence of lifestyle [10,11]. Accordingly, with a decrease in the endogenous synthesis of vitamin D_3_, the main role in covering the vitamin D requirement has been passed to a properly balanced diet and supplementation. It is worth noting that a balanced diet can cover the requirement in probably only 20% of cases [12].

In clinical practice, plasma or serum 25(OH)D_3_ concentration is measured to indicate vitamin D status in the human body. However, plasma or serum 25-hydroxyvitamin D (25(OH)D) (comprising the sum of 25(OH)D_2_ and 25(OH)D_3_) concentration is better for assessing vitamin D status because it reflects combined dietary supply and dermal production [13]. 25(OH)D_3_ has a relatively long half-life of 15–25 days (compared to 1,25(OH)_2_D_3_), a higher affinity for carrier proteins (DBP—vitamin D Binding Protein) and, consequently, a higher biological activity. Furthermore, the concentration of 1,25(OH)_2_D_3_ does not reflect vitamin D deficiency because of short half-life (3.5–21 h), low supply of precursors and insufficient endogenous synthesis, which do not cause significant changes in the concentrations of this metabolite, even over many weeks [14,15].

The problem of insufficient supply of vitamin D also concerns the Polish population. Approximately 80% of the Polish people have vitamin D deficiency [16,17].

The relationship between vitamin D concentration and diet was focused on many studies [18,19,20,21,22]. However, very few data exist on the plasma vitamin D concentration in people on the low-carbohydrate-high-fat diet [23,24].

This study assessed daily food rations, nutritional status, and plasma 25(OH)D_3_ and 25(OH)D_2_ concentration in two studied groups. The first group consisted of participants on the typical Eastern European (EE) diet, the second group on the low-carbohydrate-high-fat (LCHF) diet.

In the first part of the study, the hypothesis was verified that diet (EE vs LCHF) impacts vitamin D consumption in the studied groups. Subsequently, it was investigated if the level of vitamin D intake, reflected by plasma 25(OH)D_3_ and 25(OH)D_2_ concentration, was affected by diet in both groups.

## 2. Materials and Methods

### 2.1. Study Design and Data Collection

**Figure 1:**
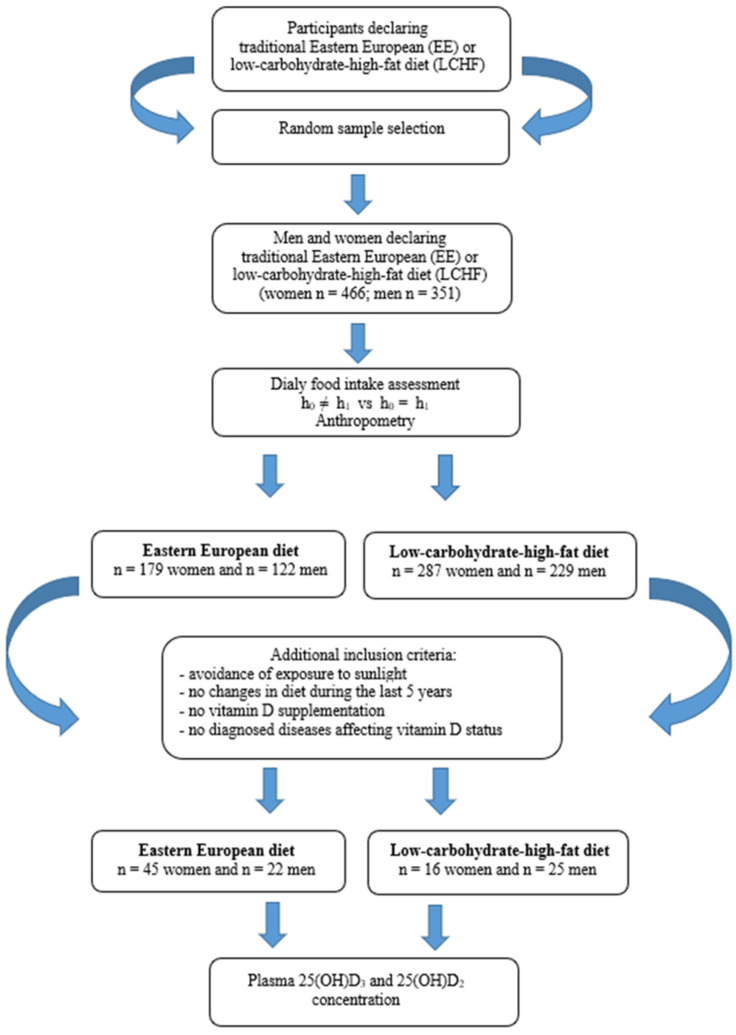
Flow diagram of study enrollment.

The study was conducted from October 2017 to March 2018 (in Poland autumn and winter period) and consisted of two stages which are presented in Figure 1. The participants were recruited by advertisements in the local media based on random selection (simple dependent randomization). In the stage I recruited volunteers declaring a traditional East European diet or LCHF diet. The type of diet declared by the participants were verified by a dietitian. The analyzed criteria were: percentage of energy from protein, fat and carbohydrate in the daily food rations (LCHF diet 1:[2.5–3.5]:[0.3–0.5]). Data analysis revealed that in the group of 466 women, 179 were on the EE diet, while 287 women used the LCHF diet. In the group of 351 men, 122 were on the EE diet and 229 on the LCHF diet. For this group, the criterion of representativeness was adopted—the relative precision of estimation not exceeding 5% for the analyzed nutrition parameters.

Stage II included assessment of plasma vitamin D concentration. In this stage, based on the participants declarations, we qualified people who met the inclusion criteria. Additional criteria in the second stage were: avoidance of exposure to sunlight, no changes in diet during the last 5 years, no vitamin D supplementation, no diagnosed diseases affecting vitamin D status.

Criteria were fulfilled by the group of 45 women and 22 men eating traditionally (on the EE diet) and 16 women and 25 men on the LCHF diet. In this group, plasma 25(OH)D_3_ and 25(OH)D_2_ concentration was determined.

People participating in this study were informed about the purpose of the research, anonymity, and the option of voluntary participation. Patients expressing a willingness to participate in the research and fulfilling the criteria signed the necessary statements, including consent to blood sampling.

The study was approved by the institutional Bioethics Committee at the Poznan University of Medical Science (Resolution, No. 422/17) and by all appropriate administrative bodies. The study was conducted in accordance with ethical standards laid down in the relevant version of the Declaration of Helsinki.

### 2.2. Assessment of Nutrition and Anthropometric Measures

A self-reported 3-day estimated food record (including one weekend day) was applied to assess food intake [25]. Food consumption was estimated based on information verified by a certified dietician with a face-to-face nutritional interview. The standardized food photo album was used to help evaluate the quantity of food consumed. Nutritional data were collected daily from each patient and were logically and substantively verified by a dietician. The energy value of the diet, consumption, and percentage of energy from protein, fat and carbohydrate in the daily food ratio and the estimated vitamin D intake was determined using the certificated dietary software (Diet 6.0, Warsaw, Poland). Food group consumption was determined, assuming 12 groups of products. The sine qua non-condition, confirming the regular diet based on a LCHF diet, was the percentage of energy from fat above 65% and carbohydrate below 20% in the daily food ratio.

Anthropometric measurements, including body weight and height, were taken according to current recommendations. The body mass index (BMI) was calculated as body weight divided by the square of body height (kg/m^2^) [26].

### 2.3. Determination of Vitamin D Metabolites

Separation of 25(OH)D_2_ and 25(OH)D_3_ was performed on a UHPLC Nexera set coupled with an LCMS-8030 triple-quadrupole tandem mass spectrometer detector (both from Shimadzu, Tokyo, Japan). Briefly, 200 μL of plasma was spiked with 20 μL of internal standard solution (0.5 μg/mL of 25(OH)D_3_-d6) and 200 μL of water was added. Protein precipitation was performed by 400 μL of methanol. Then, 800 μL of hexane was added, mixed for 3 min and centrifuged for 10 min (3500 g, 20 °C). 600 μL of the organic layer was evaporated at 45 °C. The resulting residue was dissolved in 200 μL of methanol-water solution (80:20, *v*/*v*), and 10 μL was injected onto the HPLC—MS/MS system. The analytes were separated in the Kinetex F5 analytical column (50 mm × 2.1 mm, 2.6 μm) connected to a security guard cartridge (both from Phenomenex, Torrance, CA, USA). The mobile phase was composed of methanol and water (80:20, *v*/*v*), containing 0.1% formic acid, flowed at a rate of 0.35 mL/min. The eluent from the HPLC column was introduced directly to the MS interface using electrospray ionization in the positive ion mode. MS parameters were as follows: interface temperature 350 °C, DL temperature 250 °C, heat block temperature 400 °C, nebulizing gas flow 2 L/min and drying gas flow 15 L/min. The electrospray needle voltage was 4.5 kV.

The method was linear in the concentration range of 1–100 ng/mL. The precision of the method, expressed by the relative standard deviation, was 16.3%. Accuracy described as the relative error of determinations was 14.8%. Recovery of 25(OH)D_2_ and 25(OH)D_3_ from human plasma was about 70%. The analytes proved to be stable during the storage conditions and sample pretreatment [27].

### 2.4. Statistical Analysis

The statistical analysis was performed using the Statistica software (v. 13.0), Tulsa, OH, USA. Descriptive statistics were used to describe the basic nutritional parameters and plasma vitamin D concentration of people’s diets on the EE diet and LCHF diet. Mean values and standard deviations were calculated. Additionally, the tables provide information about estimated relative precision (ς). The normality of the distribution was analyzed with the Shapiro-Wilk test. The significance of differences between the variables was tested (in normal distributions) with the Student’s t-test, while in the cases of variables where distributions deviated from the normal distribution, the Mann-Whitney U test. The statistical power of the tests was calculated to assess the minimum effect size. The level of significance was assumed at *p* < 0.05.

## 3. Results

The characteristics of the studied groups were presented in Table 1. No significant differences were observed in age, body mass, or body height between participants on the EE diet and the LCHF diet, both in men and women groups (*p* > 0.05). However, men on the EE diet had a statistically higher BMI than men on the LCHF diet (*p* < 0.05). In the case of women, we did not observe this relation. The relative precision of the analyzed anthropometric parameters (ς%) ranged from 0.29% to 2.36%.

The estimated total energy intake in daily food rations was lower in men on a LCHF diet (*p* < 0.05). In contrast, in women, total energy intake was similar regardless of the diet. Daily protein intake, regardless of the diet, was similar (*p* > 0.05). The percentage of energy from protein was 12–13%, irrespective of the diet and sex. Statistically significant differences were observed in daily fat and carbohydrate intake (*p* < 0.05). Fat consumption in daily LCHF food rations was twice higher than in EE food rations in the men and women groups. The percentage of energy from fat was: the women EE group: 32.2 ± 8.8%; the women LCHF group: 68.3 ± 8.5%; the men EE group: 33.4 ± 8.6%; the men LCHF group: 69.6 ± 8.7%. Carbohydrate consumption was three times lower in the LCHF diet. Total vitamin D intake in daily food rations varied by diet (women EE: 3.22 ± 7.16 µg; women LCHF: 8.13 ± 6.52 µg; men EE: 5.90 ± 10.9 µg; men LCHF: 9.91 ± 8.61 µg) (*p* < 0.05). The relative precision of ς (%) estimated for the analyzed nutrient parameters ranged from 1.45% to 22.1%.

Plasma total 25(OH)D_3_ and 25(OH)D_2_ concentration of the studied groups was presented in Table 2. 

Plasma 25(OH)D_3_ concentration was significantly higher in the group on the LCHF diet than in the group on the EE diet (LCHF: 34.9 ± 15.9 ng/mL; EE: 22.6 ± 12.1 ng/mL, *p* < 0.05). Plasma 25(OH)D_2_ concentration was lower than 25(OH)D_3_ concentration. However, there were no statistical differences between participants eating traditionally and participants on the LCHF diet (7.64 ± 2.42 ng/mL and 8.71 ± 3.51 ng/mL, respectively; *p* > 0.05). In epidemiologic studies, 25(OH)D_2_ and 25(OH)D_3_ are usually not analyzed separately, and the total concentration of 25(OH)D metabolites is measured.

Estimated total vitamin D metabolites (25(OH)D_2_ plus 25(OH)D_3_) plasma concentration was higher in the LCHF group (47.7 ± 17.7 ng/mL) than in the EE group (28.0 ± 10.7 ng/mL) (*p* < 0.05). The relative precision of the estimation of plasma concentration of vitamin D ranged from 4.33% to 7.07%.

Total plasma 25(OH)D_2_ and 25(OH)D_3_ concentration in women and men is presented in Table 3.

Regardless of the type of diet and sex, the dominant metabolite of vitamin D was 25(OH)D_3_ (*p* < 0.05). However, the 25(OH)D_3_ concentration was significantly higher in participants on the LCHF diet both in the women and men groups (women LCHF: 37.8 ± 16.2 ng/mL; women EE: 22.9 ± 11.9 ng/mL; men LCHF: 33.0 ± 15.9 ng/mL; men EE 22.1 ± 12.8 ng/mL). No significant differences were observed in plasma 25(OH)D_2_ between participants on the EE and LCHF diet in the men and women groups (*p* > 0.05). The precision of the results ranged from 2.84% to 7.92%. It is worth noting that plasma 25(OH)D_2_ concentration in the group of women was higher than in the group of men, regardless of the type of diet.

**Figure 2:**
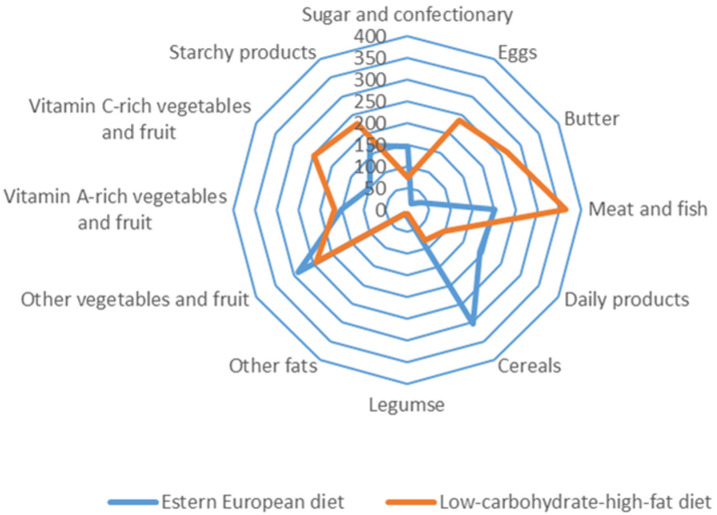
Food group consumption of 12 dietary groups among Eastern European and low-carbohydrates-high-fat subjects [g/24 h].

The consumption of 12 dietary food groups in the study subjects is illustrated in Figure 2.

Participants on the LCHF diet and the EE diet were characterized by various consumption of food groups. Subjects on the traditional diet (EE) consumed mainly cereals, meat and fish, dairy products, vegetables, and fruit. In contrast, subjects on the LCHF diet consumed mainly meat and fish offal, butter, eggs, fruit, vegetables and starchy foods.

## 4. Discussion

Despite a varied and balanced diet, dietary vitamin D intake is often unable to cover the body’s needs. This is due to the vitamin D content in food products being very low. Exceptions are fish and fish products and, to a lesser extent, butter, cream, egg yolk, liver, and mushrooms [28,29,30].

80% of vitamin D requirements are met by endogenous synthesis in the skin under UV radiation and impacted by time and date, weather, latitude, air pollution, and UV filters [31,32].

Accordingly, vitamin D deficiency is still a worldwide epidemic and clinical problem. Many countries recommend vitamin D supplementation and fortify some food products [28,29]. However, there is insufficient scientific evidence that a change to the typical EE model of nutrition can improve vitamin D status, excluding the population where there is a high consumption of fish and fish products [33,34,35,36].

This study compared two different free-living groups depending on diet. The reference point was the all-day food rations and plasma concentration of vitamin D metabolites in people on a traditional diet (Eastern European) and people on the LCHF diet. The participants have been on the diets for more than five years.

An analysis of the anthropometric parameters in both groups of women (EE vs LCHF) and both groups of men (EE vs LCHF) did not show significant differences. However, in the group of men, the BMI value was significant. Moreover, the BMI value in the group of men on the EE and LCHF diet indicated overweight (Table 1). All assessed anthropometric parameters were characterized by good estimation precision. The higher BMI values in the EE group of men seems to be explained by the level of energy intake and protein, fat and carbohydrate content (Table 1). The estimated total energy intake was lower in the LCHF group, especially in men (*p* < 0.05). This result seems to confirm the observations of other authors where the beneficial effects of the LCHF diet support reducing body weight [37,38,39,40,41]. The LCHF diet is characterized by a high content of dietary fat, while at the same time, its carbohydrate content is low. In this diet, the main source of energy for the nervous system cells and others that use glucose as an energy source is the endogenous synthesis of ketone bodies [42]. The estimated fat and carbohydrate intake (percentage of energy) between the study groups (EE vs LCHF) was statistically different, thus meeting the criteria of the study (*p* < 0.05). At the same time, the percentage of energy from protein was similar in both groups (EE vs LCHF) (*p* > 0.05). It is worth noting that the difference in the intake of energy components contributed to the content of vitamin D in the diet. The deficiency of this component is still observed in the Polish population, where supplementation is recommended in the winter months, but preferably throughout the year [42].

Participants on the LCHF diet consumed more vitamin D than those on the EE diet, especially from animal products—cholecalciferol and from plant products—ergocalciferol. This indicates that a change in diet may increase more than two times the content of these components in the diet. This difference is due to the change in the consumption structure. The LCHF diet, compared to the usual EE diet, is rich in meat and meats products, butter, eggs, cream, cheese, which are characterized by a high-fat content, and at the same time is poor in vegetables and fruits or cereals [43,44]. These differences were presented in Figure 2, which shows the food group consumption of 12 dietary groups in the studied subjects. The criterion for assessing the frequently used qualitative diet usually uses division into 12 dietary groups. On the other hand, the main sources of vitamin D in the LCHF group were eggs, offal, fatty fish and their products. The analysis of the standard deviation value and the estimation precision for this component indicates the “nutritional regimen” in the LCHF group, which results in less variation in the levels of consumption of products that are a source of vitamin D. Unfortunately, the relative precision of estimation and high variability in the vitamin D intake with all-day food rations were above 5%.

The habitual use of the LCHF diet remains controversial [44,45,46,47]. The high consumption of fats and animal products, which are the source of saturated fatty acids and dietary cholesterol, leads to a higher risk of diseases, especially cardiovascular type [38,46].

The purpose of this study is to critically analyze the differences in the level of vitamin D intake resulting from various food group consumption and its effect on the plasma vitamin D metabolite concentration. According to current knowledge, the level of vitamin D in the body is reflected by the total concentration of its hydroxyl metabolites, 25(OH)D_3_ and 25(OH)D_2_, which is a stable parameter that does not undergo rapid changes due to endogenous skin synthesis and diet. Table 2 compares plasma vitamin D concentration in the LCHF and EE groups. The differences between the numbers of participants in this table were associated with available 25(OH)D_3_ and 25(OH)D_2_ results. The 25(OH)D_3_ metabolite was detected in all EE (*n* = 67) and LCHF (*n* = 41) subjects. The 25(OH)D_2_ metabolite was detected only among *n* = 23 EE subjects, and *n* = 14 LCHF subjects, vitamin D concentration in the other samples was below the limit of quantification. Therefore, total metabolite concentration (D_3_ + D_2_) was estimated in a group of 14 subjects.

Plasma 25(OH)D_3_ concentration was significantly higher in people on the LCHF diet. Simultaneously the differences in plasma 25(OH)D_2_ concentration between the LCHF vs EE groups were insignificant (*p* > 0.05). The observation may be caused by the higher consumption of products rich in vitamin D_3_ in the LCHF group than in the EE group while the consumption of vitamin D_2_ reach products were at similar level. However, this hypothesis needs further study. Moreover, vitamin D_2_ is much less effective than vitamin D_3_ in raising plasma 25(OH)D (comprising the sum of 25(OH)D_2_ and 25(OH)D_3_). This is believed to be due to a lesser affinity of 25(OH)D_2_ to the vitamin D binding protein than that of 25(OH)D_3_, resulting in more rapid clearance of 25(OH)D_2_ [6]. Differences in total concentration of vitamin D metabolites (D_3_ + D_2_) were significant between the studied groups, and the concentration was almost twice as high in the LCHF group. The high relative precision in estimating plasma vitamin D concentration, connected with the high power of the test confirming significant differences, increases the credibility of the presented research results.

Circulating concentration of 25(OH)D is used diagnostically and clinically as well as in the derivation of dietary reference values for vitamin D [48]. There is a lack of data supporting the long-term efficacy, safety and health benefits of the LCHF diet, especially with results of serum vitamin D concentration. The LCHF diet may probably be used in patients at increased risk of vitamin D deficiency, with consideration of the risk for both gains and losses. The recent study by Alathari et al. has shown an association of metabolic, genetic risk scores with higher BMI and lower 25(OH)D concentration and has identified a novel interaction of vitamin D genetic risk scores with carbohydrate intake on body fat percentage. In this study, women with increased genetic risk of vitamin D deficiency and consuming higher amounts of carbohydrate had increased body fat composition [23]. In addition, another study confirmed that vitamin D supplementation is more effective when given with fat-containing food [24].

Table 3 presents the plasma 25(OH)D_3_ and 25(OH)D_2_ concentration separately in the group of women and men. The results confirmed that the concentration of the 25(OH)D_3_ was statistically higher in the LCHF group when compared with the EE group (*p* < 0.05), both in the women and men. In contrast, the concentration of 25(OH)D_2_ did not show significant differences (*p* > 0.05), although, in the LCHF group, they were on average 1/3 higher.

The difference in vitamin D concentration between sexes is probably related to the amount of body fat mass and its distribution [49,50].

The presented results have practical and cognitive aspects, with both strengths and limitations. The limitations of the presented study results include different numbers of subjects in the groups in which the plasma concentration of vitamin D metabolites was determined. The random nature of the sample selection for the study was assumed, which resulted in high values of intra-group variance and, consequently, differentiated precision of the estimation of individual variables. It is necessary to increase the sample size from the general population to improve the parameters of statistical evaluation.

There is no doubt that the difference in the plasma 25(OH)D_3_ concentration, and to a lesser extent the 25(OH)D_2_ concentration, were a derivative of a different diet. The endogenous synthesis of vitamin D metabolites and factors influencing its course and vitamin D supplementation were nearly identical in both groups and had a minor effect on metabolite levels. Further studies are needed to determine D_2_ and D_3_ intake separately, because the applied computer program Diet 6.0 enabled an estimation of the total vitamin D intake levels. Moreover, additional factors that may impact vitamin D metabolism should be studied, including genetic polymorphisms of enzymes engaged in the metabolic activation of vitamin D. To assess a long-term influence of a diet on the vitamin D status, a prospective study would be helpful including determination of differences in 25-hydroxy-D2 and D3 concentrations at the beginning and the end of the study.

The applied research methodology concerning assessing the level of consumption of differentiating nutrients and using a modern method for the determination of vitamin D metabolites in plasma (HPLC-MS/MS) are among the strengths of the presented research results.

## 5. Conclusions

This study confirmed that the type of diet influences the concentration of vitamin D metabolites in the plasma. The LCHF diet had a positive influence on plasma vitamin D concentration. However, long-term use of the LCHF diet remains contentious due to the high risk of cardiovascular disease.

## Figures and Tables

**Table 1:** Characteristics of the study population and macronutrient intakes ^1^.

	Women			Men		
	EE (*n* = 179)	LCHF (*n* = 287)	*p ^2^*	EE (*n* = 122)	LCHF (*n* = 229)	*p ^2^*
Age, y ς, %	56.3 ± 4.8 1.25	59.7 ± 7.1 1.38	NS	57.6 ± 5.0 1.55	63.0 ± 9.7 2.01	NS
Body height, cm ς, %	163 ± 6 0.54	163 ± 5 0.33	NS	172 ± 6 0.67	173 ± 6 0.48	NS
Bodyweight, kg ς, %	67.2 ± 12.0 2.64	67.3 ± 6.6 1.14	NS	81.5 ± 13.2 2.90	77.2 ± 12.4 2.10	NS
BMI, kg/m^2^ ς, %	25.5 ± 4.6 2.63	25.3 ± 2.6 1.18	NS	27.7 ± 4.4 2.84	25.8 ± 3.2 1.61	*p* < 0.05
Macronutrient intake						
Total energy, kcal/d ς (%)	1768 ± 581 4.85	1688 ± 516 3.57	NS	2336 ± 807 6.19	2168 ± 724 4.37	*p* < 0.05
Protein, g/d ς (%)	55.8 ± 19.5 5.16	53.9 ± 17.1 3.71	NS	72.0 ± 33.2 8.26	66.9 ± 22.2 4.46	NS
Protein, % kcal ς (%)	12.9 ± 3.4 3.83	13.2 ± 2.8 2.51	NS	12.3 ± 3.1 4.52	12.8 ± 3.0 3.06	NS
Fat, g/d ς (%)	64.0 ± 30.1 * 6.94	130 ± 44.4 * 4.02	*p* < 0.05	88.6 ± 42.8 * 8.66	170 ± 62.4 * 4.80	*p* < 0.05
Fat, % kcal ς (%)	32.2 ± 8.8 * 4.03	68.3 ± 8.5 * 1.45	*p* < 0.05	33.4 ± 8.6 * 4.62	69.6 ± 8.7 * 1.63	*p* < 0.05
Carbohydrate, g/d ς (%)	242 ± 88 * 5.39	80.0 ± 34 * 4.95	*p* < 0.05	310 ± 105 * 6.07	97.4 ± 45 * 6.07	*p* < 0.05
Carbohydrate, % kcal ς (%)	55.0 ± 10.0 * 2.68	19.6 ± 6.4 * 3.80	*p* < 0.05	53.1 ± 9.2 * 3.09	18.3 ± 6.4 * 4.54	*p* < 0.05
Vitamin D intake, µg/d ς (%)	3.22 ± 7.16 * 19.1	8.13 ± 6.52 * 9.37	*p* < 0.05	5.90 ± 10.9 * 22.1	9.91 ± 8.61 * 11.4	*p* < 0.05

^1^ Data were expressed as means ± SDs; ^2^ Statistically significant difference (*p* < 0.05); power of test = 0.95 unless otherwise indicated; * power of test = 0.35; EE—East European diet; LCHF—low-carbohydrate-high-fat diet; NS—no statistical differences.

**Table 2:** Plasma total 25(OH)D_3_ and 25(OH)D_2_ concentrations in subjects on low-carbohydrates-high-fat diet and on Eastern European diet [ng/mL].

	25(OH)D_3_		25(OH)D_2_		Total (D_3_ + D_2_)	
	EE (*n* = 67)	LCHF (*n* = 41)	EE (*n* = 23)	LCHF (*n* = 14)	EE (*n* = 14)	LCHF (*n* = 14)
Mean	22.6	34.9	7.64	8.71	28.0	47.7
SD	12.1	15.9	2.42	3.51	10.7	17.7
ς(%)	7.07	6.08	4.33	5.75	5.45	5.29
*p* value	0.00004 *		0.27987		0.00304 **	

EE—East European diet, LCHF—low-carbohydrate-high-fat diet; 25(OH)D_3_—25-hydroxyvitamin D_3_; 25(OH)D_2_—25-hydroxyvitamin D_2_; SD—standard deviation; ς—relative precision of the result estimation; *—the power of test = 0.9; **—the power of test = 0.8.

**Table 3:** Plasma total vitamin D metabolites’ concentrations [ng/mL] among men and women under traditional Eastern European and low-carbohydrates-high-fat diet.

	Women				Men			
	25(OH)D_3_		25(OH)D_2_		25(OH)D_3_		25(OH)D_2_	
	EE (*n* = 45)	LCHF (*n* = 16)	EE (*n* = 18)	LCHF (*n* = 7)	EE (*n* = 22)	LCHF (*n* = 25)	EE (*n* = 5)	LCHF (*n* = 7)
Mea	22.9	37.8	6.83	7.89	22.1	33.0	10.6	10.1
SD	11.9	16.2	1.79	4.33	12.8	15.9	2.24	1.78
ς (%)	6.90	6.03	3.65	8.85	7.92	6.56	3.86	2.84
*p* value	0.0003 *		0.7853		0.0034		0.8709	

EE—East European diet, LCHF—low-carbohydrate-high-fat diet; 25(OH)D_3_—25-hydroxyvitamin D_3_; 25(OH)D_2_—25-hydroxyvitamin D_2_; SD—standard deviation; ς—relative precision of the result estimation; *—the power of test = 0.9.

## Data Availability

Data available on request due to restrictions privacy.
